# The genome sequence of the tapered dronefly, *Eristalis pertinax* (Scopoli, 1763)

**DOI:** 10.12688/wellcomeopenres.17267.2

**Published:** 2021-12-21

**Authors:** William Hawkes, Karl Wotton, University of Oxford and Wytham Woods Genome Acquisition Lab, Darwin Tree of Life Barcoding collective, Wellcome Sanger Institute Tree of Life programme, Wellcome Sanger Institute Scientific Operations: DNA Pipelines collective, Tree of Life Core Informatics collective, Darwin Tree of Life Consortium

**Affiliations:** 1 Centre for Ecology and Conservation, University of Exeter, Penryn, Cornwall, UK

**Keywords:** Eristalis pertinax, tapered dronefly, genome sequence, chromosomal

## Abstract

We present a genome assembly from an individual male *Eristalis pertinax *(the tapered dronefly; Arthropoda; Insecta; Diptera; Syriphidae). The genome sequence is 487 megabases in span. The majority of the assembly (95.23%) is scaffolded into seven chromosomal pseudomolecules, with the X and Y sex chromosomes assembled. The complete mitochondrial genome was also assembled and is 17.2 kilobases in length.

## Species taxonomy

Eukaryota; Metazoa; Ecdysozoa; Arthropoda; Hexapoda; Insecta; Pterygota; Neoptera; Endopterygota; Diptera; Brachycera; Muscomorpha; Syrphoidea; Syrphidae; Eristalinae; Eristalini; Eristalis; *Eristalis pertinax* (Scopoli, 1763) (NCBI:txid1572519).

## Introduction

The tapered dronefly, *Eristalis pertinax*, is a fairly large hoverfly separated from others in the Eristalis genus by the presence of yellow tarsi on their front and middle legs. *E. pertinax* mimics the general shape and colouring of a honeybee, *Apis mellifera*, to gain protection against bird predation through Batesian mimicry. Their mimicry of their model species extends beyond simple colouration, as *E. pertinax* has been shown to spend similar times foraging and flying to that of *Apis mellifera* ( Golding & Edmunds, 2000), as well as acoustic mimicry through ‘defence buzzes’ ( Moore & Hassall, 2016).

*E. pertinax* is widespread in the British Isles, occurring in a range of habitats, perhaps favouring woodlands and wetlands. They can be found on the wing between March and November. Throughout the year, *E. pertinax* shows seasonal polyphenism through two distinct morphs (a larger, long-haired morph in the spring, and a smaller, short haired morph in the summer), presumably adaptive to the different seasonal temperatures ( Mielczarek *et al.*, 2016).

Male *E. pertinax* hoverflies are highly territorial, defending sunny patches of woodland rides or gardens where females are likely to rest or forage for food. These hoverflies feed on nectar from a wide variety of flowers, but hogweed ( *Heracleum* sp.) and bramble ( *Rubus* sp.) are thought to be their preferences ( Herkenrath, 2014).

Their larvae are colloquially known as rat-tailed maggots and live in a wide array of organically rich pools. The larvae feed on decaying organic matter and therefore play a highly important ecological role in terms of decomposition ( Hurtado *et al.*, 2008).

This is the first production of a high-quality *E. pertinax* genome; we believe that the sequence described here, generated as part of the Darwin Tree of Life project, will further aid understanding of the biology and ecology of this hoverfly.

## Genome sequence report

**Figure 1.**
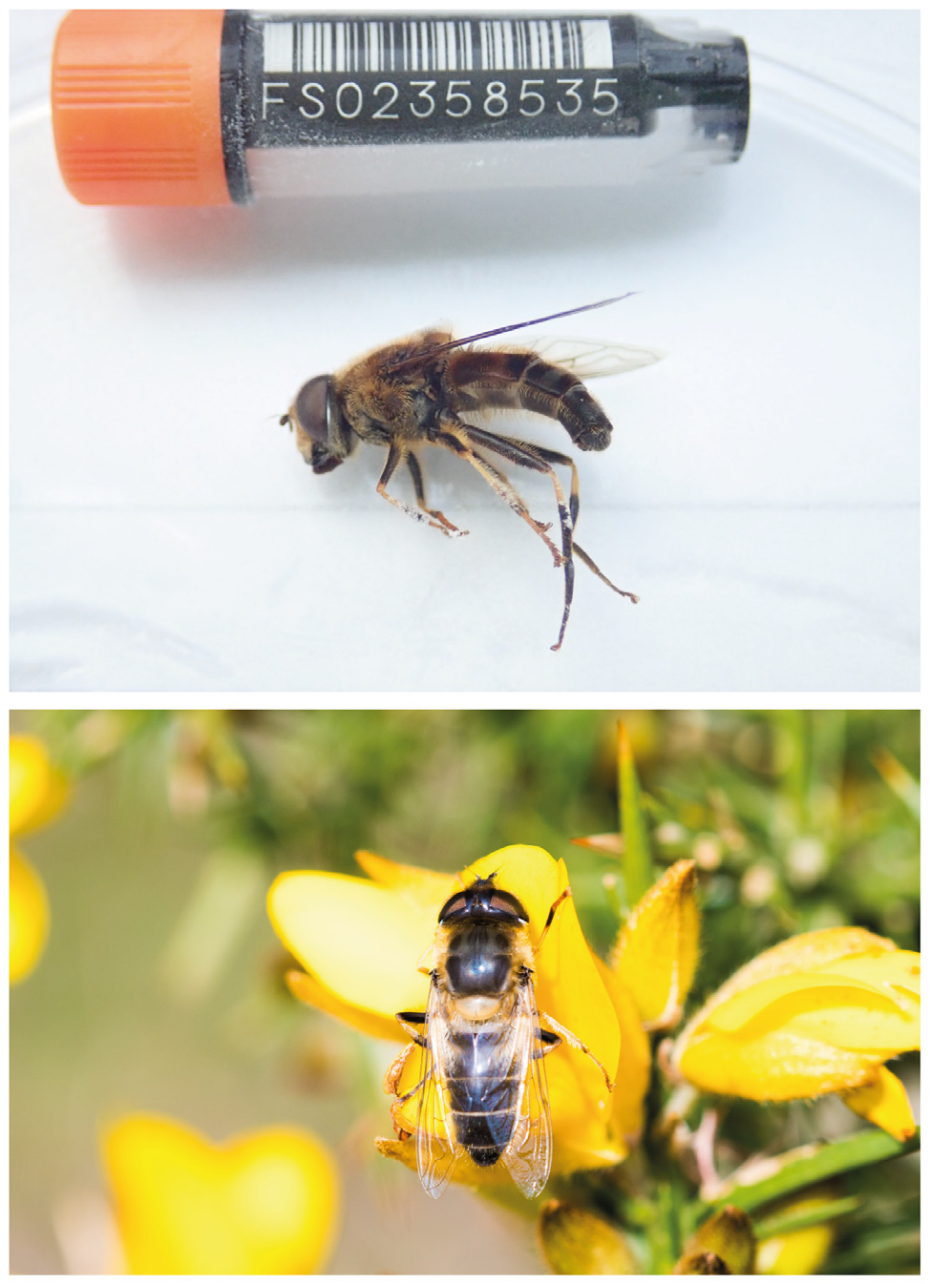
Images of *Eristalis pertinax*. Above, image of the idEriPert2 specimen used in genome sequencing, taken during preservation and processing; below, example image taken by William Hawkes of a live female *E. pertinax*.

The genome was sequenced from a single male *E. pertinax* collected from Wytham Great Wood, Oxfordshire, UK (latitude 51.772, longitude -1.339) (see Figure 1 for an example photograph of *E. pertinax*). A total of 29-fold coverage in Pacific Biosciences single-molecule long reads and 73-fold coverage in 10X Genomics read clouds were generated. Primary assembly contigs were scaffolded with chromosome conformation Hi-C data. Manual assembly curation corrected 181 missing/misjoins, reducing the scaffold number by 32.45%, and increasing the scaffold N50 by 185.38%.

**Figure 2.**
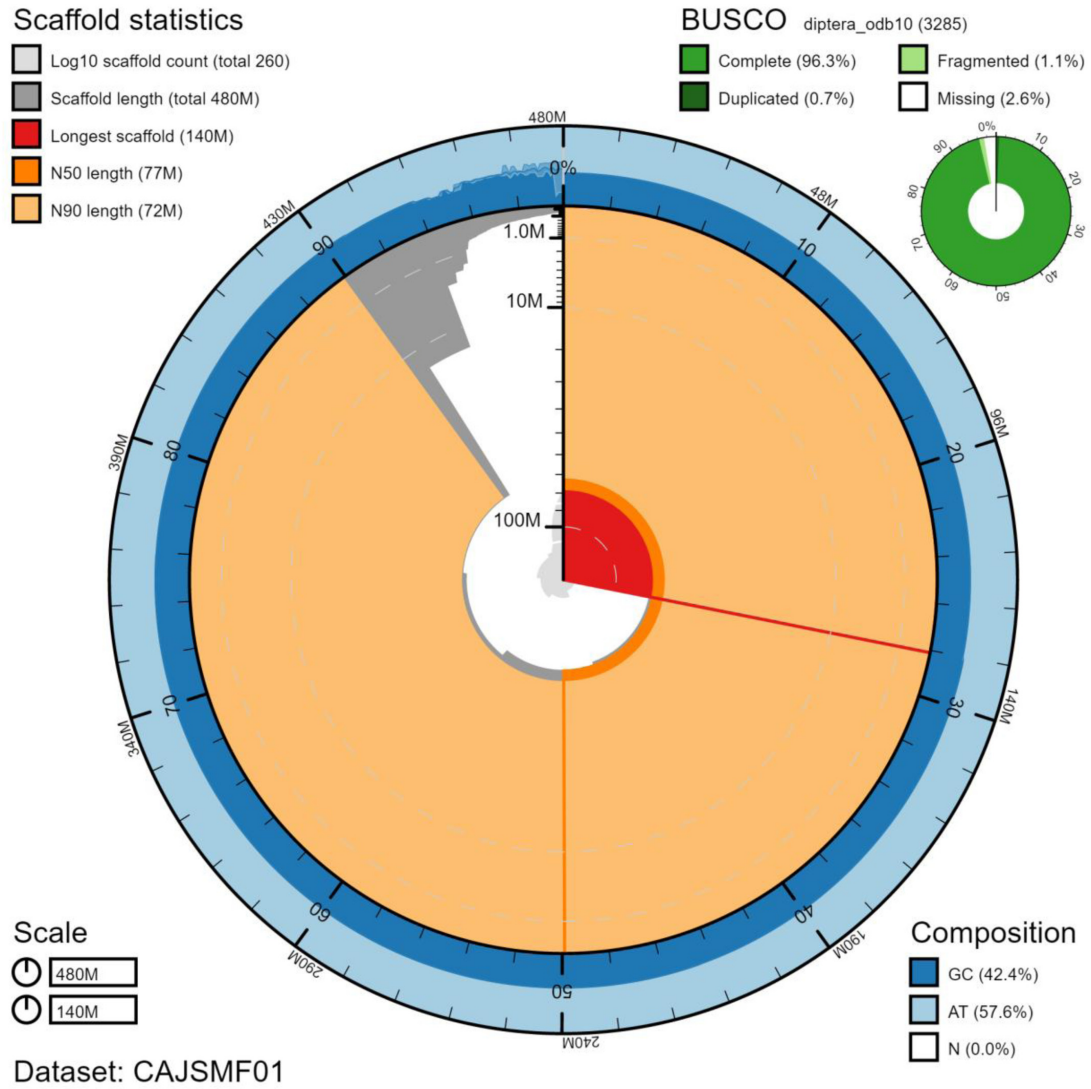
Genome assembly of *Eristalis pertinax*, idEriPert2.1: metrics. The BlobToolKit Snailplot shows N50 metrics and BUSCO gene completeness. The main plot is divided into 1,000 size-ordered bins around the circumference with each bin representing 0.1% of the 482,092,357 bp assembly. The distribution of scaffold lengths is shown in dark grey with the plot radius scaled to the longest chromosome present in the assembly (135,593,448 bp, shown in red). Orange and pale-orange arcs show the N50 and N90 scaffold lengths (77,495,269 and 72,030,237 bp), respectively. The pale grey spiral shows the cumulative scaffold count on a log scale with white scale lines showing successive orders of magnitude. The blue and pale-blue area around the outside of the plot shows the distribution of GC, AT and N percentages in the same bins as the inner plot. A summary of complete, fragmented, duplicated and missing BUSCO genes in the diptera_odb10 set is shown in the top right. An interactive version of this figure is available at https://blobtoolkit.genomehubs.org/view/idEriPert2.1/dataset/CAJSMF01/snail.

The final assembly has a total length of 482 Mb in 257 sequence scaffolds with a scaffold N50 of 77.5 Mb ( Table 1). The majority, 95.23%, of the assembly sequence was assigned to seven chromosomal-level scaffolds, representing five autosomes (numbered by sequence length), and the X and Y sex chromosomes ( Figure 2– Figure 5; Table 2). The assembly has a BUSCO v5.1.2 ( Simão *et al.*, 2015) completeness of 96.3% using the diptera_odb10 reference set. While not fully phased, the assembly deposited is of one haplotype. Contigs corresponding to the second haplotype have also been deposited.

**Table 1.** Genome data for *Eristalis pertinax*, idEriPert2.1.

*Project accession data*	
Assembly identifier	idEriPert2.1
Species	*Eristalis Pertinax*
Specimen	idEriPert2 (PacBio, 10X, Hi-C); idEriPert1 (RNAseq)
NCBI taxonomy ID	219539
BioProject	PRJEB43008
BioSample ID	SAMEA7520160
Isolate information	Male, head/thorax, abdomen (idEriPert2); female, abdomen (idEriPert1)
*Raw data accessions*	
PacificBiosciences SEQUEL II	ERR6560799
10X Genomics Illumina	ERR6054732-ERR6054735
Hi-C Illumina	ERR6054736
Illumina PolyA RNAseq	ERR6054737
*Genome assembly*	
Assembly accession	GCA_907269125.1
*Accession of alternate* * haplotype*	GCA_907269085.1
Span (Mb)	482
Number of contigs	574
Contig N50 length (Mb)	3.5
Number of scaffolds	257
Scaffold N50 length (Mb)	77.5
Longest scaffold (Mb)	135.6
BUSCO * genome score	C:96.3%[S:95.7%,D:0.7%], F:1.1%,M:2.6%,n:3285

*BUSCO scores based on the diptera_odb10 BUSCO set using v5.1.2. C= complete [S= single copy, D=duplicated], F=fragmented, M=missing, n=number of orthologues in comparison. A full set of BUSCO scores is available at https://blobtoolkit.genomehubs.org/view/idEriPert2.1/dataset/CAJSMF01/busco.

**Figure 3.**
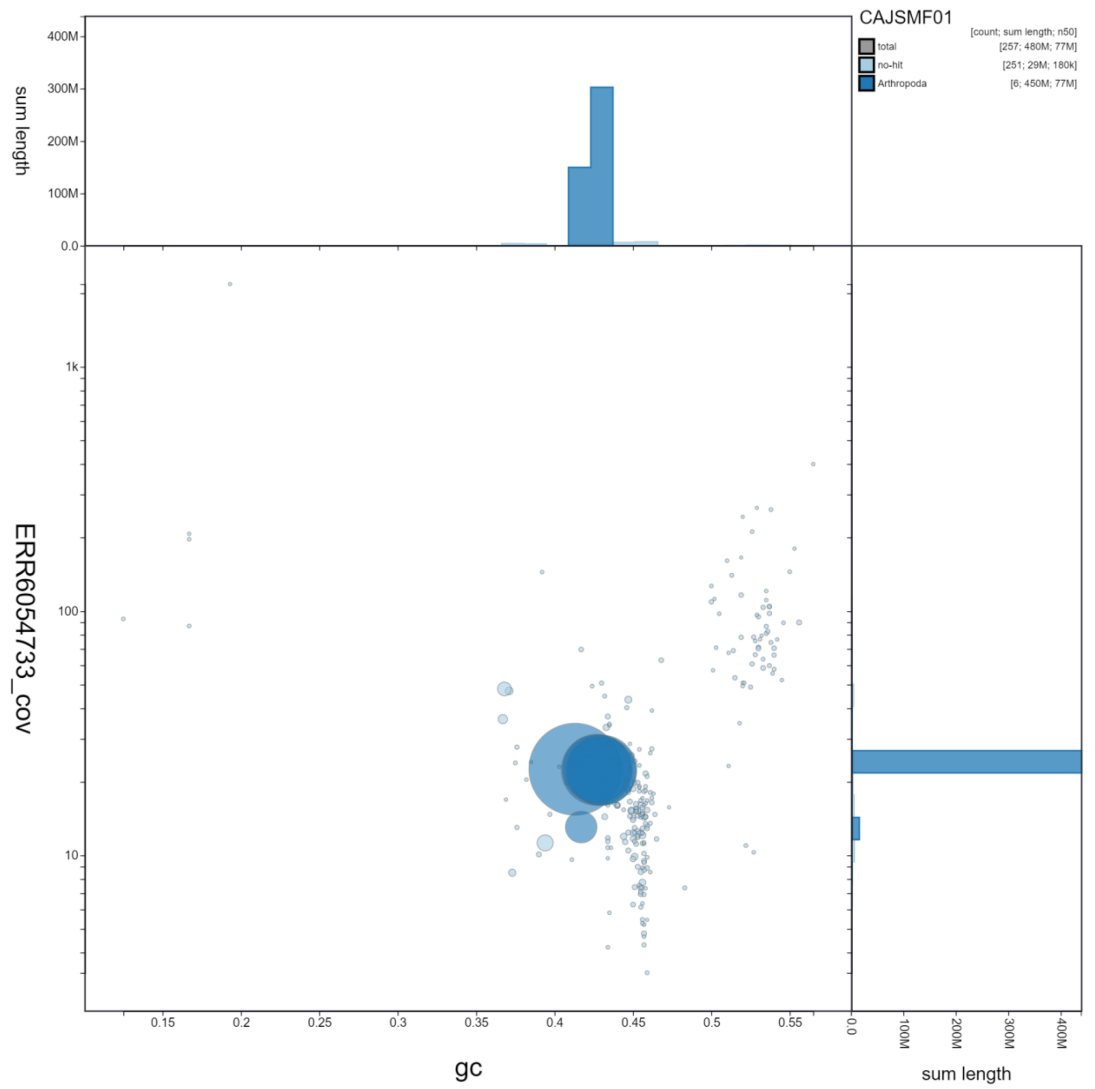
Genome assembly of *Eristalis pertinax*, idEriPert2.1: GC coverage. BlobToolKit GC-coverage plot. Scaffolds are coloured by phylum. Circles are sized in proportion to scaffold length. Histograms show the distribution of scaffold length sum along each axis. An interactive version of this figure is available at https://blobtoolkit.genomehubs.org/view/idEriPert2.1/dataset/CAJSMF01/blob.

**Figure 4.**
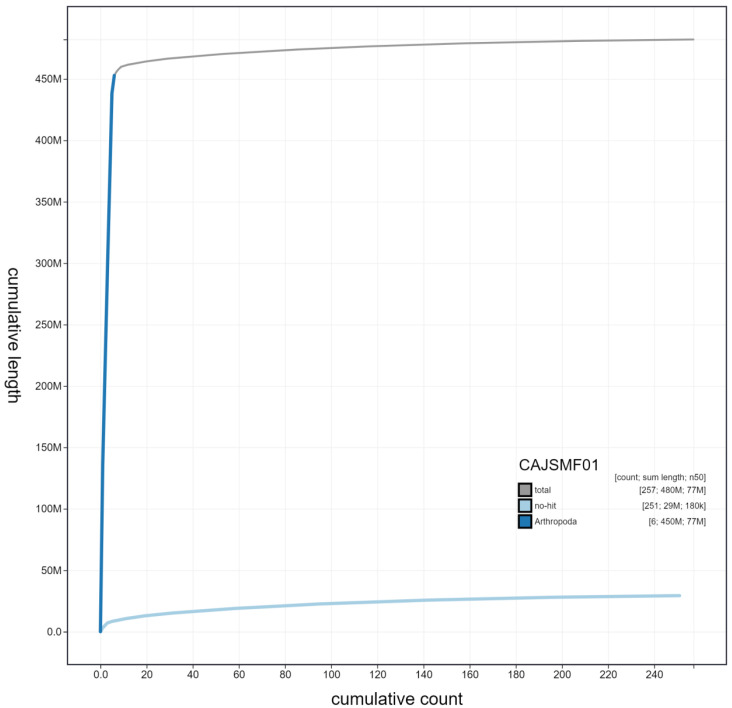
Genome assembly of *Eristalis pertinax*, idEriPert2.1: cumulative sequence. BlobToolKit cumulative sequence plot. The grey line shows cumulative length for all scaffolds. Coloured lines show cumulative lengths of scaffolds assigned to each phylum using the buscogenes taxrule. An interactive version of this figure is available at https://blobtoolkit.genomehubs.org/view/idEriPert2.1/dataset/CAJSMF01/cumulative.

**Figure 5.**
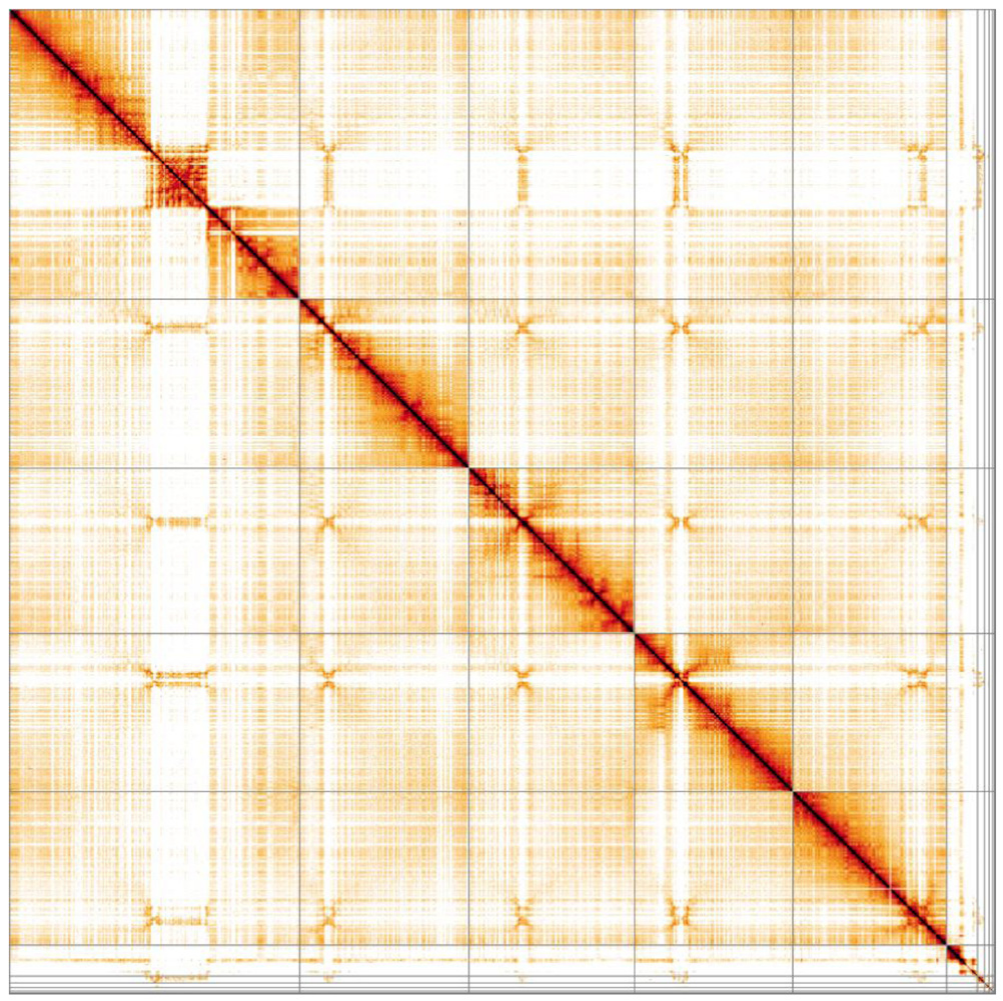
Genome assembly of *Eristalis pertinax*, idEriPert2.1: Hi-C contact map. Hi-C contact map of the idEriPert2.1 assembly, visualised in HiGlass. Chromosomes are arranged in size order from left to right and top to bottom.

**Table 2.** Chromosomal pseudomolecules in the genome assembly of *Eristalis pertinax*, idEriPert2.1.

INSDC accession	Chromosome	Size (Mb)	GC%
OU026145.1	1	135.59	41.3
OU026146.1	2	79.22	42.7
OU026147.1	3	77.5	43
OU026148.1	4	73.96	43
OU026149.1	5	72.03	42.7
OU026150.1	X	14.45	41.7
OU026151.1	Y	3.19	39.4
OU026152.1	MT	0.02	18.8
-	Unplaced	26.15	44

## Methods

A male (idEriPert2) and a female (idEriPert1) *E. pertinax* sample were collected from Wytham Great Wood, Oxfordshire, UK (latitude 51.772, longitude -1.339) by Will Hawkes, University of Exeter on 1 August 2019 (idEriPert2) and 7 August 2019 (idEriPert1). The specimens were caught with a net, snap-frozen on dry ice and stored using a CoolRack.

DNA was extracted at the Tree of Life laboratory, Wellcome Sanger Institute (WSI). The idEriPert2 sample was weighed and dissected on dry ice with tissue set aside for Hi-C sequencing. Head/thorax tissue was cryogenically disrupted to a fine powder using a Covaris cryoPREP Automated Dry Pulveriser, receiving multiple impacts. Fragment size analysis of 0.01–0.5 ng of DNA was then performed using an Agilent FemtoPulse. High molecular weight (HMW) DNA was extracted using the Qiagen MagAttract HMW DNA extraction kit. Low molecular weight DNA was removed from a 200-ng aliquot of extracted DNA using 0.8X AMpure XP purification kit prior to 10X Chromium sequencing; a minimum of 50 ng DNA was submitted for 10X sequencing. HMW DNA was sheared into an average fragment size between 12–20 kb in a Megaruptor 3 system with speed setting 30. Sheared DNA was purified by solid-phase reversible immobilisation using AMPure PB beads with a 1.8X ratio of beads to sample to remove the shorter fragments and concentrate the DNA sample. The concentration of the sheared and purified DNA was assessed using a Nanodrop spectrophotometer and Qubit Fluorometer and Qubit dsDNA High Sensitivity Assay kit. Fragment size distribution was evaluated by running the sample on the FemtoPulse system.

RNA was extracted from abdomen tissue of idEriPert1 in the Tree of Life Laboratory at the WSI using TRIzol (Invitrogen), according to the manufacturer’s instructions. RNA was then eluted in 50 μl RNAse-free water and its concentration assessed using a Nanodrop spectrophotometer and Qubit Fluorometer using the Qubit RNA Broad-Range (BR) Assay kit. Analysis of the integrity of the RNA was done using Agilent RNA 6000 Pico Kit and Eukaryotic Total RNA assay.

Pacific Biosciences HiFi circular consensus and 10X Genomics Chromium read cloud sequencing libraries were constructed according to the manufacturers’ instructions. Poly(A) RNA-Seq libraries were constructed using the NEB Ultra II RNA Library Prep kit. Sequencing was performed by the Scientific Operations core at the Wellcome Sanger Institute on Pacific Biosciences SEQUEL II (HiFi), Illumina HiSeq X (10X) and Illumina HiSeq 4000 (RNA-Seq) instruments. Hi-C data were generated from abdomen tissue of idEriPert2 using the Arima v2 Hi-C kit in the Tree of Life laboratory and sequenced at the Scientific Operations core on HiSeq X.

Assembly was carried out with Hifiasm ( Cheng *et al.*, 2021); haplotypic duplication was identified and removed with purge_dups ( Guan *et al.*, 2020). One round of polishing was performed by aligning 10X Genomics read data to the assembly with longranger align, calling variants with freebayes ( Garrison & Marth, 2012). The assembly was then scaffolded with Hi-C data ( Rao *et al.*, 2014) using SALSA2 ( Ghurye *et al.*, 2019). The assembly was checked for contamination and corrected using the gEVAL system ( Chow *et al.*, 2016) as described previously ( Howe *et al.*, 2021). Manual curation was performed using gEVAL, HiGlass ( Kerpedjiev *et al.*, 2018) and Pretext. The mitochondrial genome was assembled using MitoHiFi ( Uliano-Silva *et al.*, 2021), which performed annotation using MitoFinder ( Allio *et al.*, 2020). The genome was analysed and BUSCO scores generated within the BlobToolKit environment ( Challis *et al.*, 2020). Table 3 contains a list of all software tool versions used, where appropriate.

**Table 3.** Software tools used.

Software tool	Version	Source
Hifiasm	0.12	Cheng *et al.*, 2021
purge_dups	1.2.3	Guan *et al.*, 2020
SALSA2	2.2	Ghurye *et al.*, 2019
longranger align	2.2.2	https://support.10xgenomics.com/genome-exome/ software/pipelines/latest/advanced/other-pipelines
freebayes	1.3.1-17-gaa2ace8	Garrison & Marth, 2012
MitoHiFi	1	Uliano-Silva *et al.*, 2021
gEVAL	N/A	Chow *et al.*, 2016
HiGlass	1.11.6	Kerpedjiev *et al.*, 2018
PretextView	0.1.x	https://github.com/wtsi-hpag/PretextView
BlobToolKit	2.6.2	Challis *et al.*, 2020

## Data availability

European Nucleotide Archive: Eristalis pertinax (tapered dronefly). Accession number PRJEB44981; https://identifiers.org/ena.embl/PRJEB44981.

The genome sequence is released openly for reuse. The *E. pertinax* genome sequencing initiative is part of the Darwin Tree of Life (DToL) project. All raw sequence data and the assembly have been deposited in INSDC databases. The genome will be annotated using the RNA-Seq data and presented through the Ensembl pipeline at the European Bioinformatics Institute. Raw data and assembly accession identifiers are reported in Table 1.

## References

[ref-20] Allio R Schomaker-Bastos A Romiguier J : MitoFinder: Efficient automated large-scale extraction of mitogenomic data in target enrichment phylogenomics. *Mol Ecol Resour.* 2020;20(4):892–905. 10.1111/1755-0998.13160 32243090 PMC7497042

[ref-1] Challis R Richards E Rajan J : BlobToolKit - Interactive Quality Assessment of Genome Assemblies. *G3 (Bethesda).* 2020;10(4):1361–74. 10.1534/g3.119.400908 32071071 PMC7144090

[ref-2] Cheng H Concepcion GT Feng X : Haplotype-Resolved de Novo Assembly Using Phased Assembly Graphs with Hifiasm. *Nat Methods.* 2021;18(2):170–75. 10.1038/s41592-020-01056-5 33526886 PMC7961889

[ref-3] Chow W Brugger K Caccamo M : gEVAL — a Web-Based Browser for Evaluating Genome Assemblies. *Bioinformatics.* 2016;32(16):2508–10. 10.1093/bioinformatics/btw159 27153597 PMC4978925

[ref-4] Garrison E Marth G : Haplotype-Based Variant Detection from Short-Read Sequencing.arXiv: 1207. 2012;3907. Reference Source

[ref-5] Ghurye J Rhie A Walenz BP : Integrating Hi-C Links with Assembly Graphs for Chromosome-Scale Assembly. *PLoS Comput Biol.* 2019;15(8):e1007273. 10.1371/journal.pcbi.1007273 31433799 PMC6719893

[ref-6] Golding YC Edmunds M : Behavioural Mimicry of Honeybees ( *Apis Mellifera*) by Droneflies (Diptera: Syrphidae: *Eristalis* Spp.). *Proc Biol Sci.* 2000;267(1446):903–9. 10.1098/rspb.2000.1088 10853733 PMC1690622

[ref-7] Guan D McCarthy SA Wood J : Identifying and Removing Haplotypic Duplication in Primary Genome Assemblies. *Bioinformatics.* 2020;36(9):2896–98. 10.1093/bioinformatics/btaa025 31971576 PMC7203741

[ref-8] Herkenrath P : The Hoverflies (Syrphidae) of Fen Drayton Lakes. *Plate 1 Fenland Flora Coverage Mid-December 2013 How Many Species Recorded since 2006?* 2014.

[ref-9] Howe K Chow W Collins J : Significantly Improving the Quality of Genome Assemblies through Curation. *GigaScience.* 2021;10(1):giaa153. 10.1093/gigascience/giaa153 33420778 PMC7794651

[ref-10] Hurtado P Pérez-Bañón C Gladis T : Biology of Saprophagous Hoverflies (Diptera, Syrphidae) and Its Role in Degrading of Pig Slurry. In *XXIII International Congress of Entomology, Durban (South Africa)*.2008.

[ref-11] Kerpedjiev P Abdennur N Lekschas F : HiGlass: Web-Based Visual Exploration and Analysis of Genome Interaction Maps. *Genome Biol.* 2018;19(1):125. 10.1186/s13059-018-1486-1 30143029 PMC6109259

[ref-12] Mielczarek LE Oleksa A Meyza K : Seasonal Polyphenism in *Eristalis Pertinax* (Diptera: Syrphidae). *Eur J Entomol.* 2016;113:489–96. 10.14411/eje.2016.064

[ref-13] Moore CD Hassall C : A Bee or Not a Bee: An Experimental Test of Acoustic Mimicry by Hoverflies. *Behav Ecol: Official Journal of the International Society for Behavioral Ecology.* 2016;27(6):1767–74. 10.1093/beheco/arw107

[ref-14] Rao SSP Huntley MH Durand NC : A 3D Map of the Human Genome at Kilobase Resolution Reveals Principles of Chromatin Looping. *Cell.* 2014;159(7):1665–80. 10.1016/j.cell.2014.11.021 25497547 PMC5635824

[ref-15] Simão FA Waterhouse RM Ioannidis P : BUSCO: Assessing Genome Assembly and Annotation Completeness with Single-Copy Orthologs. *Bioinformatics.* 2015;31(19):3210–12. 10.1093/bioinformatics/btv351 26059717

[ref-16] Uliano-Silva M Nunes JGF Krasheninnikova K : marcelauliano/MitoHiFi: mitohifi_v2.0.2021. 10.5281/zenodo.5205678

