# Supplementary material for: The genome sequence of the tapered dronefly, Eristalis pertinax (Scopoli, 1763)
Source: Wellcome Open Res. 2021 Dec 21;6:292. Originally published 2021 Oct 29. [Version 2] doi: 10.12688/wellcomeopenres.17267.2 (PMC9975435; doi:10.12688/wellcomeopenres.17267.2)
Supplement: Peer review history

# Peer review: Reviewer response for version 2

**DOI:** 10.21956/wellcomeopenres.19392.r53696

**Published:** 2022-12-14

**Authors:** Markus Friedrich

**Affiliations:** 1 Department of Biological Sciences, Wayne State University, Detroit, MI, USA

This manuscript reports on the genome draft for the biologically highly interesting and, therefore, significant dipteran species Epistrophe pertinax. Data generation and analysis are exemplary.

 Minor points:

1. "This is the first production of a high-quality E. pertinax genome; we believe that the sequence described here, generated as part of the Darwin Tree of Life project, will further aid understanding of the biology and ecology of this hoverfly."
  I would separate the two sentences by a regular period.
2. "a minimum of 50 ng DNA was submitted for 10X sequencing..."
  Why not state the actual amount?
3. A sentence about whether gene order in the mitochondrial genome was conserved or different from other Diptera would be useful.

Are sufficient details of methods and materials provided to allow replication by others?

Yes

Is the rationale for creating the dataset(s) clearly described?

Yes

Are the datasets clearly presented in a useable and accessible format?

Yes

Are the protocols appropriate and is the work technically sound?

Yes

Reviewer Expertise:

Comparative genomics

I confirm that I have read this submission and believe that I have an appropriate level of expertise to confirm that it is of an acceptable scientific standard.

# Peer review: Reviewer response for version 1

**DOI:** 10.21956/wellcomeopenres.19085.r46882

**Published:** 2021-11-03

**Authors:** Jerry Lanfear

**Affiliations:** 1 ELIXIR Hub, Hinxton, UK

This paper describes the generation of a high quality, chromosome level genome of the tapered dronefly ( *Eristalis pertinax). *The layout, strategy and methods adopt the approach used by the Tree of Life Project for several other recently sequenced species. It is clear to understand what's been done within this species and to compare across other species, so it makes a lot of sense. Overall this is a quality paper and merits approval for publication.

 That said, I think there is a typographical error, which must be corrected prior to publication: In the section "Data availability" the second sentence refers to the genome of *G. alexis*, I think this should say *E. pertinax?*  

 I also have a couple of minor suggestions for the introduction: 

- First, the ID characters and distribution data could be referenced e.g. to the guidebook,  Britain's Hoverflies (Ball and Morris) or to the UK Hoverfly Recording Scheme.
- Second, it might be useful to put *E. pertinax* in context with other members of the *Eristalis* genus, and any genome data that has been generated previously (for instance, I think there is some genome data available for *E. dimidiata*, a North American species?).  It's not made clear that this is not just the first published high quality genome for *E. pertinax* but I think for any species in this important genus.

Are sufficient details of methods and materials provided to allow replication by others?

Yes

Is the rationale for creating the dataset(s) clearly described?

Yes

Are the datasets clearly presented in a useable and accessible format?

Yes

Are the protocols appropriate and is the work technically sound?

Yes

Reviewer Expertise:

Bioinformatics, Biodiversity

I confirm that I have read this submission and believe that I have an appropriate level of expertise to confirm that it is of an acceptable scientific standard.
